# Direct Writing of Patterned, Lead‐Free Nanowire Aligned Flexible Piezoelectric Device

**DOI:** 10.1002/advs.201600120

**Published:** 2016-05-30

**Authors:** Meng Gao, Lihong Li, Wenbo Li, Haihua Zhou, Yanlin Song

**Affiliations:** ^1^ Key Laboratory of Green PrintingInstitute of ChemistryChinese Academy of SciencesBeijing100190P. R. China; ^2^ School of Chemistry and Chemical EngineeringUniversity of Chinese Academy of SciencesBeijing100049P. R. China

**Keywords:** alignment, alkaline niobate nanowires, direct writing, micropattern, piezoelectric nanogenerator

## Abstract

**A high‐performance flexible piezoelectric nanogenerator (PNG)** is fabricated by a direct writing method, which acquires both patterned piezoelectric structure and aligned piezoelectric nanowires simultaneously. The voltage output of the as‐prepared PNG is nearly 400% compared with that of the traditional spin‐coated device due to the effective utilization of stress. This facile printing approach provides an efficient strategy for significant improvement of the piezoresponse.

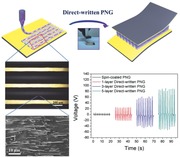

Due to the energy crises in the past decades,^1^, ^2^, ^3^, ^4^, ^5^ emerging researches have focused on harvesting the most common and accessible mechanical energy (such as human activity, vehicle rolling, and air vibration) based on piezoelectric nanogenerators (PNGs).^6^, ^7^, ^8^, ^9^, ^10^ To achieve high‐output PNGs, endeavors have been employed in exploring anisotropic piezoelectric materials such as nanowires^11^, ^12^, ^13^, ^14^, ^15^, ^16^ and nanosheets,^17^, ^18^ which show more outstanding performance compared with the traditionally used isotropic ones.^19^, ^20^, ^21^, ^22^, ^23^, ^24^ Among such materials, lead‐free (Na,K)NbO_3_ (KNN) nanowires (NWs) with remarkable piezoelectric property and biocompatibility^25^, ^26^, ^27^, ^28^ serve as one of the most attractive candidates, which are seldom studied in flexible PNG fabricating till now. Despite the pursuit of noteworthy anisotropic piezoelectric materials, highly oriented arrangement of the anisotropic piezoelectric materials^29^, ^30^, ^31^ and patterned structure of the device's piezoelectric layer,^32^, ^33^ both of which can significantly improve the electrical output of the PNGs, are also highly required. Nevertheless, most researchers manufacture PNGs by a commonly utilized spin coating method^34^, ^35^, ^36^, ^37^ which sets up obstacles for these PNGs to reach the requirement mentioned above, thus impairs their output performance. Currently, approaches such as electrospinning,^38^, ^39^ chemical epitaxial growth,^40^, ^41^ and template based lithography^42^, ^43^, ^44^ are developed to fabricate PNGs with aligned piezoelectric materials or patterned structures. However, these approaches are either complicated or confined to specific materials. Hence, there is a clear and urgent need to exploit a facile way for high‐output anisotropic piezoelectric material based PNG fabricating.

Recently, printing technology is experiencing a striking development in diverse architecture manufacturing and device fabricating.^45^, ^46^, ^47^, ^48^ Among them, direct writing which deposits continuous filament serves as a flexible approach to pattern materials for both planar and freestanding 3D structure in a layer‐by‐layer sequence.^48^, ^49^, ^50^ Moreover, the ink experiences a high shear environment within the nozzle during the printing process, which produces highly orientation of the anisotropic materials.^51^

Herein, we demonstrate a high‐performance flexible KNN NWs based PNG fabricated by a direct writing method, which acquires both micropatterned structure and aligned nanowires simultaneously. These two features both integrated in a PNG device would significantly enhance the effective utilization of stress and contribute to the strain enlargement, which consequently improves the piezoresponse. The output voltage of this direct‐written PNG is nearly 400% compared with that of the traditional spin‐coated one. In addition, by further increasing printed layers, significant performance improvement can be achieved via the multilayer printing. Furthermore, the device can successfully harvest electric energy for generating remarkable signals and driving electronic units. This direct writing strategy largely improves the output of PNGs, with great potential in fields such as self‐powered sensors and portable/wearable personal electronics.

The schematic diagram of the PNG fabrication process is shown in **Figure** **1**a and detailed information is described in the Experimental Section. Briefly, a patterned piezoelectric lamina with aligned KNN NWs is fabricated by directly writing the piezoelectric nanocomposite (p‐NC) on a bottom electrode. Then, a top electrode is attached to it. In order to orient the electric dipoles in the same direction, PNG poling is required thereafter.

**Figure 1:**
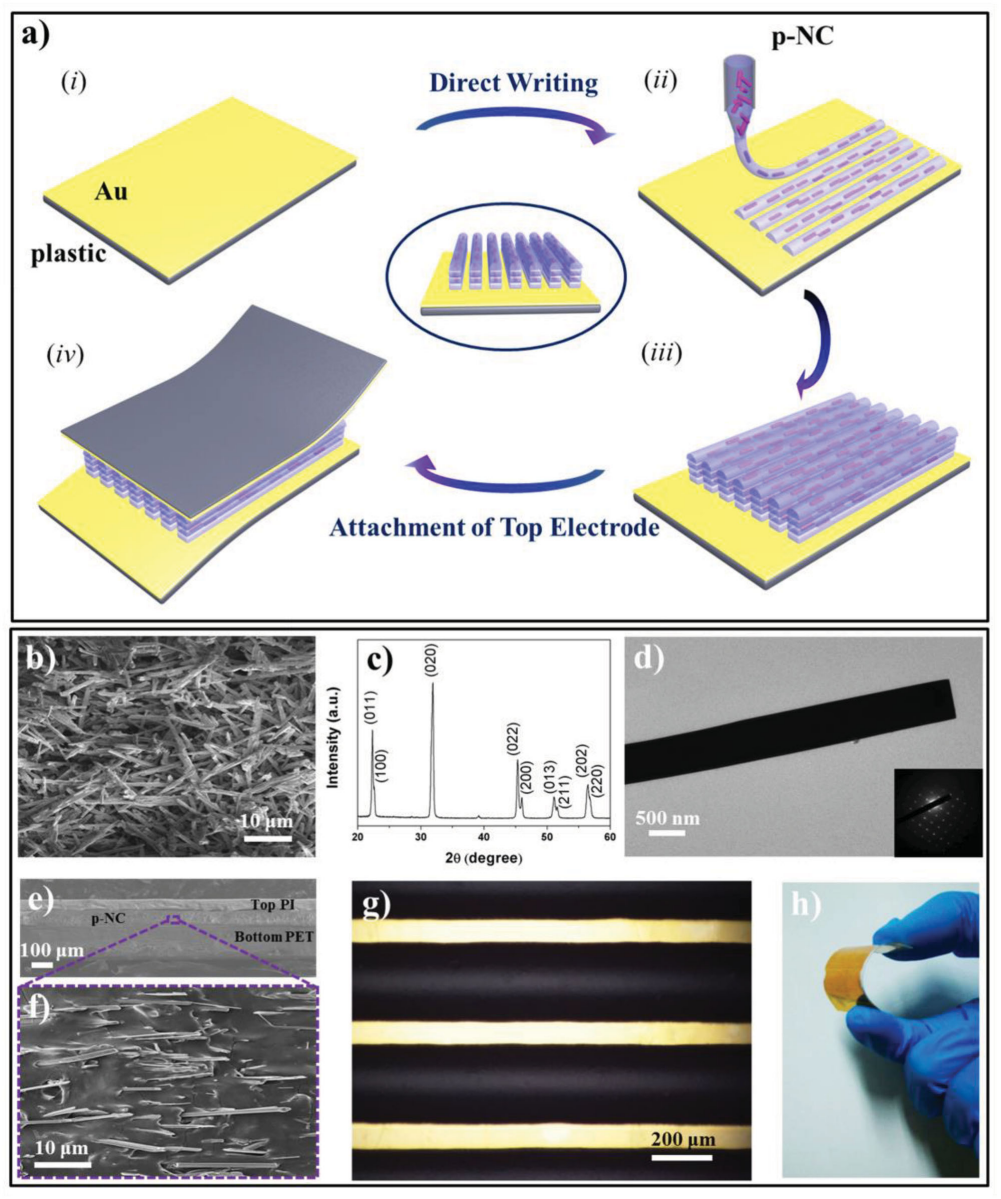
a) Schematic illustration of a multilayer patterned PNG with aligned nanowires fabricated on (i) a bottom electrode by (ii, iii) direct writing the p‐NC, followed by (iv) attachment of a top electrode. The middle inset depicts the side view of the direct‐written structure. b) SEM image, c) XRD, and d) TEM of the KNN NWs synthesized by molten‐salt synthesis method. The inset shows the corresponding SAED pattern. e) Cross‐sectional SEM image of a PNG device. f) Magnified cross‐sectional SEM photograph of the piezoelectric layer of the PNG device. g) Top optical image of the patterned piezoelectric layer fabricated by direct writing. h) Photograph of a flexible PNG bent by fingers.

The lead‐free KNN NWs used in this study are fabricated by the modified molten‐salt synthesis method (details see the Supporting Information). They generate piezoelectric potential under external stress and act as energy generation source. Scanning electron microscopy (SEM), transmission electron microscopy (TEM), X‐ray diffraction (XRD), and energy dispersive spectroscopy (EDS) are utilized to characterize the KNN NWs. The SEM image (Figure 1b) exhibits a well‐defined nanowires' morphology with an average length about 20 μm. Figure 1c shows the XRD pattern that confirms the orthorhombic structure of the synthesized KNN product, which is in agreement with the result reported before.^28^ The corresponding TEM image (Figure 1d) and the selected‐area electron‐diffraction (SAED) pattern (inset of Figure 1d) provide a more comprehensive analysis on their crystalline structure, and demonstrate the single‐crystalline nature of the KNN NWs. The K/Na ratio of the material measured by EDS is 1:1 (Figure S1, Supporting Information) which brings about the maximum of the alkaline niobate's piezoelectric coefficient d_33_ due to the morphotropic phase boundary.^25^ The relevant characterizations of the precursors (KNb_3_O_8_, H_3_ONb_3_O_8_, Nb_2_O_5_) which serve as self‐sacrificing nanowire templates for the final products KNN NWs are showed in Figure S2 (Supporting Information). KNN nanoparticles (NPs) are also fabricated to compare with their corresponding nanowires (Figure S3, Supporting Information).

Figure 1e demonstrates the cross‐sectional SEM image of a KNN NWs based direct‐written (NWs‐DW) PNG with a sandwiched structure along the direct writing direction. A magnified cross‐sectional SEM image (Figure 1f) shows that the KNN NWs adopt strongly preferential orientations in the poly(dimethylsiloxane) (PDMS) matrix. XRD pattern (Figure S4, Supporting Information) of the NWs‐DW PNG without top electrode is also measured to verify the preferential orientation. Detailed description and explanation can be referred to the Supporting Information. Piezoelectric layer's cross‐sectional SEM graphs of a KNN NWs based spin‐coated (NWs‐SC) PNG and a KNN NPs based spin‐coated (NPs‐SC) PNG are also taken for comparison (Figure S5, Supporting Information). The cross‐sectional SEM image of the NWs‐SC PNG exhibits random oriented KNN NWs in the PDMS matrix due to the inconstant direction of the centrifugal force during the spin coating process, which is completely different from the KNN NWs' arrangement in the NWs‐DW PNG. For illustration of the direct‐written structure, an optical image of the piezoelectric layer with paralleled pattern (Figure 1g) is demonstrated. The black line which shows a stereo‐morphology is the sample, while the golden line is the uncovered Au/Cr‐coated polyethylene terephthalate (PET) bottom electrode substrate. The paralleled pattern is chosen to guarantee nanowires in different filaments aligned in the same direction. Figure 1h presents a photograph of a bendable PNG with an effective area of 2 cm × 2 cm, which indicates that the device is very flexible.

**Figure** **2** presents the printing behavior of the KNN NWs based ink and its corresponding printed morphology. The rheology of the ink should be optimized to print filaments with moderate aspect ratio, which ensures the entity of the patterned piezoelectric layer architecture and keeps the underlying layers with minimal deformation. The ink used in the study compromises KNN NWs and PDMS matrix (Figure 2a inset). The rheological behavior of the inks with solid loading of different concentration (*c*) ranging from 10%–40% is studied (KNN NWs can hardly well disperse in PDMS when *c* ≥ 50%). From Figure 2a, which provides the apparent viscosity (*η*) as a function of shear rate, the viscosity is in proportion to the ink concentration. The viscosity is dramatically increased at low shear rate when the ink concentration reaches 40%. At the same time, it also exhibits highly shear thinning behavior, which guarantees the ink flowing smoothly through the nozzle during printing. Figure 2b demonstrates their storage modulus (*G*′) and loss modulus (*G*″) as a function of shear strain. For inks with concentration below 40%, they demonstrate liquid‐like response (*G*′ < G″). On the contrary, the ink with solid loading of 40% exhibits a storage modulus plateau that exceeds loss modulus by almost an order of magnitude at strain lower than shear yield strain (≈0.05%), which therefore ensures a solid‐like nature in the quiescent state. Thus it can be concluded that among the tested inks, the one with solid loading of 40% exhibits the most desired rheological behavior due to its high viscosity under low shear rate, the shear thinning behavior and the solid‐like response (*G*′ > *G*″) under low shear strain, which is distinct compared with the inks with concentration lower than 40%.

**Figure 2:**
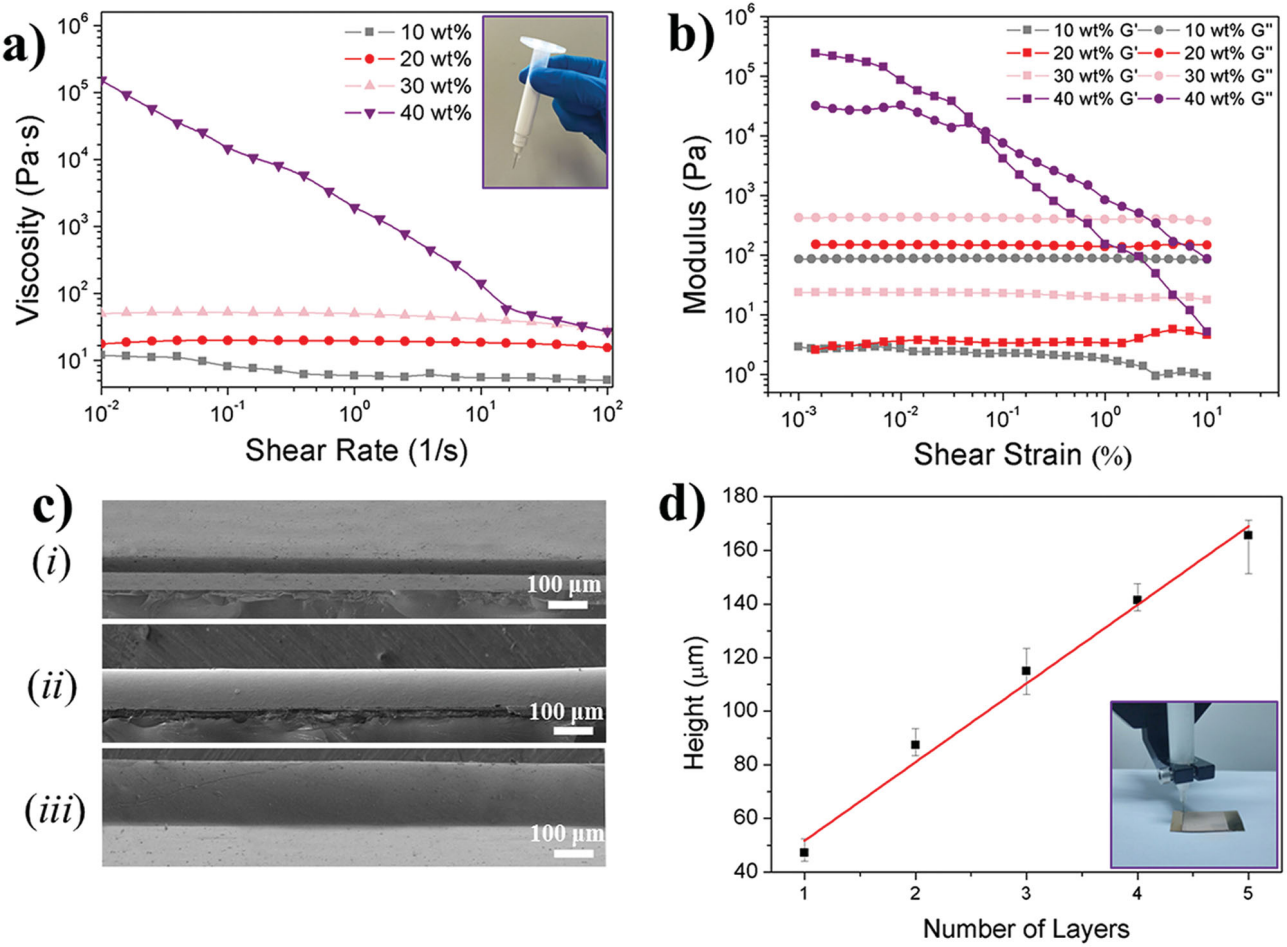
a) Ink viscosity (*η*) as a function of shear rate for the KNN NWs based inks with different concentration. The inset shows the optical image of the ink. b) Storage modulus (G′) and loss modulus (G″) as a function of shear strain. c) Side SEM images of the printed (i) 1 layer, (ii) 3 layers, (iii) 5 layers piezoelectric structure. d) Printed height features as a function of the printed layer number (200 μm nozzle diameter). The inset shows an optical image of the printing equipment.

A direct‐written printer is utilized to print the well‐performed ink through a 200 μm cylindrical nozzle (Figure 2d inset). In order to acquire a patterned PNG with enhanced piezoelectric lamina thickness, a 3D multilayer structure is printed. SEM images show the side views (Figure 2c) of 1 layer, 3 layers, and 5 layers of the piezoelectric lamina obtained through a layer‐by‐layer printing sequence, respectively. Figure 2d presents their corresponding height of filaments as a function of the printed layer number. It is obvious that the structure height enhances with the layer number. Whereas, their width remains nearly constant when the layer number increases, which is concluded from their corresponding top views and the relationship between width and the layer number (Figure S6, Supporting Information).

Generated output of the PNG device during the periodic bending/unbending tests is carried out employing a bending stage executed at a horizontal displacement of 2 cm with a bending frequency of 0.5 Hz under the 0.44% strain. It should be noted that the bending trajectory is parallel to the pattern filament's direction (*X*‐axis), which is also consistent with the nanowires' orientation. **Figure** **3**a displays three states of the mechanical motion, i.e., original, bending, and release state, and their corresponding power generation mechanism. In the bending state, the current flow is generated due to dipoles movement from equilibrium position and charges accumulation at two opposite side surfaces of the material. In the release state, the accumulated charges return to their original state, which results in output signals in the opposite direction. The 1‐layer KNN NWs‐DW PNG generates an open‐circuit voltage of ≈21.0 V and a short‐circuit current of ≈0.5 μA under mechanical deformation, respectively (Figure 3bi), and a well‐behaved, periodic alternation of negative and positive peaks of electric signals are observed. To confirm that the measured output signals are purely generated by the PNG sample, a widely used switching‐polarity test is conducted, and the inversion of voltage and current signals is demonstrated in Figure 3bii in the reverse connection. The output performance of the control device fabricated by directly writing the PDMS matrix without the KNN NWs is also measured. As shown in Figure S7 in the Supporting Information, there is no reliable signal generated from the direct‐written device containing only pure PDMS layer. The low electrical signals, which are presumably caused by several electrostatic charges at the electrodes, can be ignored in comparison with the NWs‐DW PNG.

**Figure 3:**
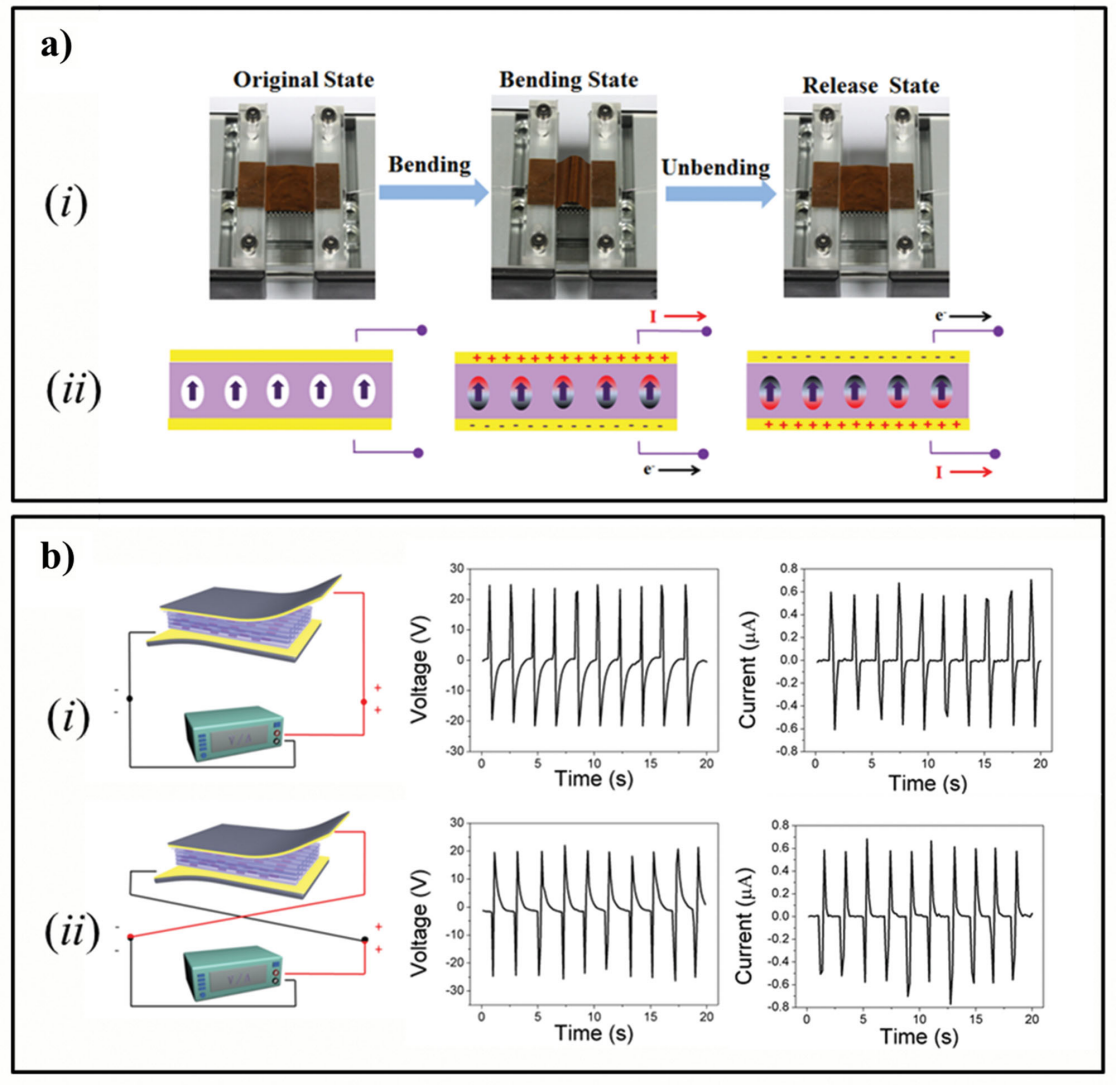
a) (i) Optical images and (ii) the schematic diagram which indicates the corresponding mechanism of the electricity generation of a PNG device in its original, bending, and release state. b) The measured open‐circuit voltage and short‐circuit current signals of a 1‐layer NWs‐DW PNG device when the measurement unit is (i) forward‐connected and (ii) reverse‐connected.

The output performance (**Figure** **4**a and Figure S8, Supporting Information) of the KNN NWs‐SC PNG with same working area and piezoelectric lamina thickness of the 1‐layer KNN NWs‐DW PNG is also measured for comparison. As shown in Figure 4a, the voltage output difference of the NWs‐SC PNG and the 1‐layer NWs‐DW PNG is apparently appreciable under identical stress. Specifically, the open‐circuit voltage of the direct‐written device (≈21.0 V) is nearly 400% compared with that of the spin‐coated one (≈5.3 V), which verifies the effect of the direct‐written structure features on the output performance of PNGs. For further confirmation, a corresponding simulation is carried out via a finite element method, and the numerical modeling result is approximately in agreement with the experimental result. The calculated piezoelectric voltage output of the NWs‐SC PNG and the 1‐layer NWs‐DW PNG is presented in Figure 4b, where piezopotential is depicted by color code. The piezoelectric lamina of the NWs‐SC PNG is a flat layer with nanowires random oriented, while the NWs‐DW PNG consists of aligned oriented nanowires located in the patterned PDMS matrix, and the same number of nanowires is included in both simulations for a fair comparison. For visual recognition of the inner nanowires' arrangement inside the PDMS matrix, half‐transparent photographs corresponding to the model in calculation are illustrated as displayed in Figure S9 (Supporting Information).

**Figure 4:**
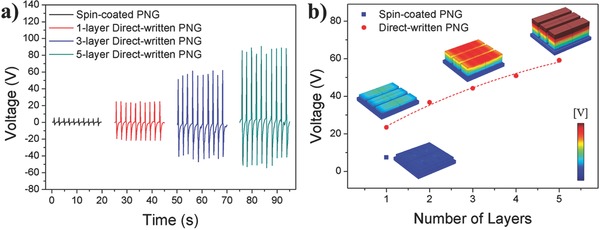
a) Experiment results of voltage output of a NWs‐SC PNG and NWs‐DW PNGs with different layers. b) Simulation results of voltage output of a NWs‐SC PNG and NWs‐DW PNGs with different layers.

The aligned nanowires and the micropatterned morphology which are both integrated in the PNG device via the direct writing method, tend to explain in‐depth the enhanced energy harvesting property. KNN NWs in the PDMS matrix adopt strongly preferential orientations (Figure 1f) after extrusion from the nozzle because of the applied shear force induced alignment.^51^ The identity of the nanowires' longitudinal direction and the force direction leads to an enlargement of the nanowires' deformation, thus results in exaggerated dipole displacement and piezoelectric property. Apart from the advantage caused by the nanowires' alignment, the micropatterned morphology with paralleled filaments may also result in the performance enhancement. When the force is applied along the nanowires' longitudinal direction (*X*‐axis), the PDMS matrix tends to deform, which lays a compression on the piezoelectric component, namely the KNN NWs. The stress would inevitably lead to strain not only in the parallel direction (*X*‐axis) but also in the perpendicular direction (*Y*‐axis) of the nanowires' orientation. However strain in *Y*‐axis is not wanted because force in the radial direction of the piezoelectric nanowires leads to less deformation than force in the longitudinal direction, thus impairing the PNG output performance. If there is a gap between filaments, the structure would restrict stress relaxation in *Y*‐axis more effectively, which accordingly increases the strain in *X*‐axis. The consistency of the nanowires' direction, pattern filaments' direction, and force direction results in an enlargement of strain in a cooperative manner. Thereby, the micropatterned piezoelectric lamina with nanowires in aligned arrangement fabricated by direct writing would significantly enhance the degree of stress sensitivity and the efficiency of mechanical impact transfer, which inevitably contributes to the strain enlargement. The open‐circuit voltage (*V*_out_) generated by the flexible PNG is interpreted as^36^, ^52^

(1) $$V_{\mathrm{out}}=\int g_{31}\cdot \varepsilon (l)\cdot Y\;\;dl$$

where *l* is the perpendicular distance between the adjacent electrodes, ε(*l*) is the function of the strain along the direction of *l*, *Y* is the Young's modulus, and *g*_31_ is the piezoelectric voltage coefficient. The increasing of strain inside the composite inevitably leads to an enhanced voltage output according to the equation. The inner mechanism of the relationship between strain variations and the piezoelectric output can be referred to the Supporting Information.

Energy harvesting property of KNN NPs‐SC PNG is also investigated (Figure S10, Supporting Information). It can be concluded that piezoelectric nanowires play a superior effect than nanoparticles in energy harvesting, which might be due to their effective transport of charge carriers, high piezoelectricity, and responsiveness to tiny random mechanical disturbances. Thus, the nanowires based PNG provides feasibility for scenarios where only small triggering forces are available and contributes to higher sensibility. The cooperative effect of material's morphology, alignment of nanowires, and the pattern of piezoelectric lamina would lead to the exaggerating performance of the piezoelectric property.

To further improve the voltage output of this well‐performed direct‐written device, PNGs with increasing piezoelectric lamina thickness realized by 3D multilayer direct writing are fabricated. The open‐circuit voltage output (Figure 4a) and the short‐circuit current output (Figure S8, Supporting Information) of NWs‐DW PNGs with 3 layers and 5 layers of the piezoelectric lamina under dynamic bending–unbending cycles are measured. As expected, it shows that the electric performance increases with respect to the layer number and consequently the distance between electrodes (*d*). The device generates high voltage output up to ≈72.2 V compared with that of previous reported lead‐free PNGs (Table S1, Supporting Information) when a 5‐layer PNG is made. Similarly, the calculated voltage output as a function of printed layer number is also performed (Figure 4b). To be mentioned, further increasing layer of the PNG is not studied because of the piezoelectric layer fracture induced by thickness enhancement.

Depending on the exceptional piezoelectric characteristic of the direct‐written PNG device, applications of sensing the mechanical movement and harvesting energy are demonstrated. When a finger with the PNG wrapped on is mechanically bent (**Figure** **5**a), apparent voltage signals and a huge amount of voltage are generated as illustrated in Figure 5b. As for the deviation of the output peaks, it is attributed to the finger's compression impacted on the device, which results in the voltage output enhancement, and the irregular speed of the finger mechanical motion. Apart from sensing mechanical movement, the power generated by the PNG is also applied to operate commercial electronic units. Commercial light‐emitting diodes (LEDs) are connected in series for power generation demonstration (Figure 5c). To store the electrical energy from periodically bending the PNG device, it is incorporated in a circuit comprising a full‐wave bridge rectifier with four diodes and a 2.2 μF capacitor connected in series as demonstrated in top inset of Figure 5d. During the charging process, the total stored voltage in the capacitor reaches up to 37.7 V (bottom inset of Figure 5d) by continual bending/unbending deformation of the PNG. The stored energy is sufficient to light up 12 commercial LED arrays in series as depicted in Figure 5d. These results demonstrate that the direct‐written PNG can successfully harvest electric energy for generating remarkable signals and driving electronic units.

**Figure 5:**
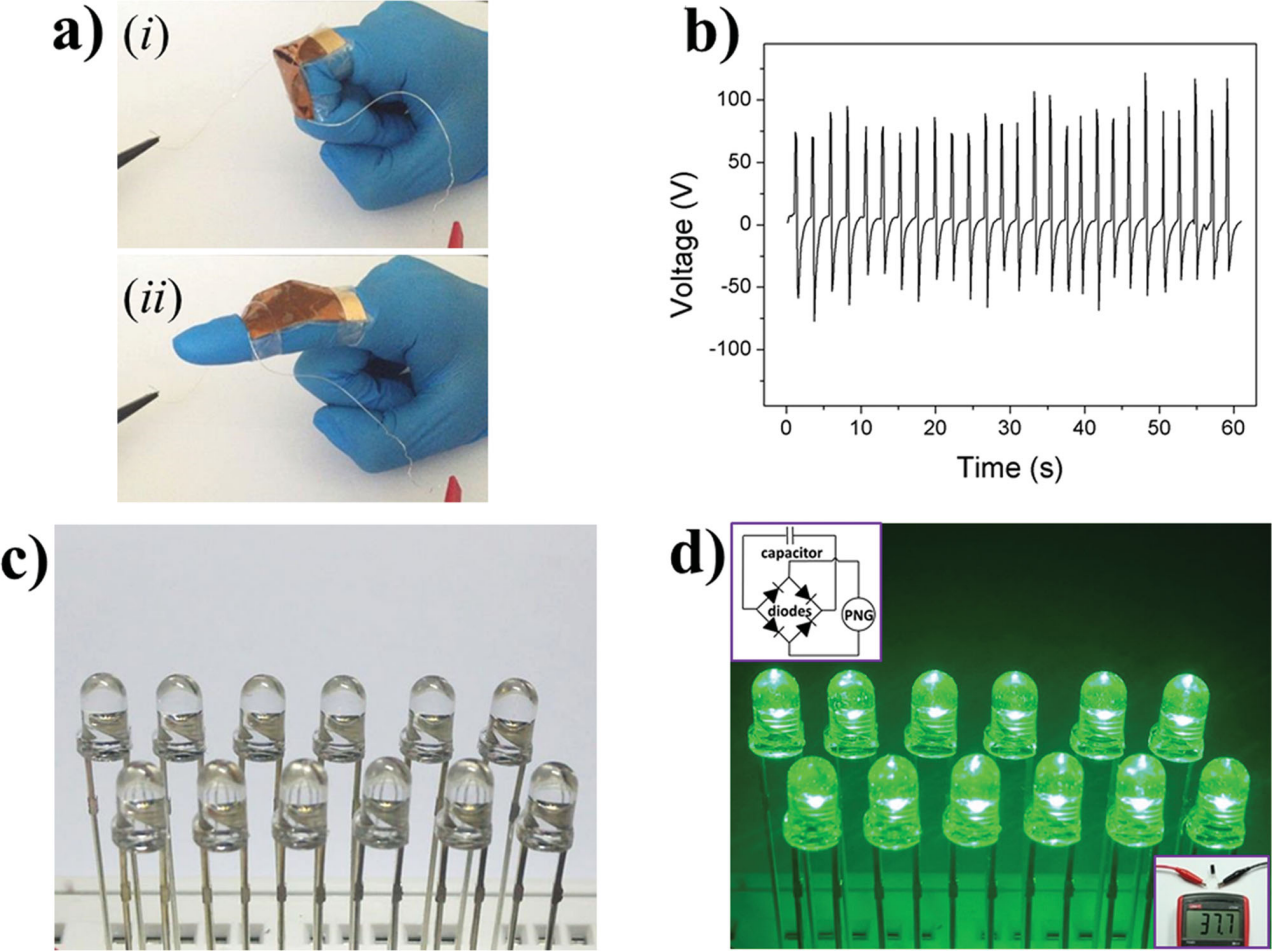
a) Photograph of the finger movement (i) bending and (ii) unbending. b) Output voltage of a 5‐layer NWs‐DW PNG device during the irregular bending of a human finger. c) A photograph taken from 12 unlighted green LEDs. d) A captured image of the LEDs driven by the capacitor charged by the PNG device under periodical bending motion. The top inset shows the schematic diagram of the rectifying and charging circuit comprising four diodes and a capacitor. The bottom inset indicates the total stored voltage in the capacitor.

A high‐performance flexible KNN NWs based PNG is fabricated by a direct writing method, which acquires both patterned piezoelectric structure and aligned piezoelectric nanowires simultaneously. The voltage output of the as‐prepared PNG is nearly 400% compared with the traditional spin‐coated one due to the consistency of nanowires' direction, pattern filaments' direction, and force direction, which exaggerates the effective utilization of stress and the induced high degree of strain. Moreover, by further increasing printed layers, significant performance improvement can be achieved with a maximum voltage output up to ≈72.2 V via the multilayer printing. This simple, effective, and nonlithographic printing approach applied to various materials provides an efficient strategy for significant improvement of the piezoresponse, allowing for usage in fields such as energy harvesting and pressure sensing. Furthermore, it can also be potentially adopted as a strategy for enhancing performance of the recently developed triboelectric nanogenerator^53^, ^54^, ^55^ by patterning the contact surface.

## Experimental Section

*Preparation of the Piezoelectric Nanocomposite Based Ink*: Piezoelectric nanocomposite‐based inks for direct writing and spin coating were prepared by dispersing 40 wt% KNN NWs (or KNN NPs) in PDMS precursor which was the mixture of PDMS base and curing agent in the proportion of 10:1 by weight. The inks were mechanically stirred at 800 rpm for 0.5 h in an icing bath for homogenization.

*Direct Writing of the Microptterned Piezoelectric Layer*: The substrate used for direct writing was the bottom electrode, namely an Au/Cr‐coated PET film (150 μm in thickness) whose conducting layer was deposited by a high vacuum thermal evaporation system (PATOR, ATT010). The KNN NWs/PDMS ink was loaded in a syringe and extruded through a micronozzle with inner diameter of 200 μm onto the substrate under an applied pressure of 30 psi at a speed of 10 mm s^−1^. The micropattern was created using a 3‐axis micropositioning stage. The micropatterned piezoelectric layer was fully cured at 150 °C for 0.5 h to retain its morphology.

*Spin Coating of the Flat Piezoelectric Layer*: The KNN NWs/PDMS (or KNN NPs/PDMS) ink was spin‐coated onto the bottom electrode at the rate of 2500 rpm (2000 rpm) for 30 s and cured at 80 °C for 2 h.

*PNG Fabrication*: Then, an Au/Cr‐coated polyimide film (50 μm in thickness) used as top electrode was attached to the surface of the piezoelectric layer with PDMS precursor served as an adhesive agent. The PDMS adhesive agent was spin‐coated onto the top electrode at the rate of 3000 rpm for 30 s and procured at 80 °C for 5 min. After fully hardened at 80 °C for 2 h, the packaged device was poled at 150 °C by applying an electric field for 12 h. 1‐layer and spin‐coated PNG were poled at 0.4 kV. 3‐layer and 5‐layer PNG were poled at 1.2 and 2.0 kV, respectively.

*Characterization and Measurements*: The SEM image was characterized using a Hitachi S‐4800 scanning electron microscope operating at 5 kV accelerating voltage. The TEM image was taken with a JEOL TEM‐2100 transmission electron microscope operating at 200 kV accelerating voltage. XRD pattern was carried out using a Rigaku D/MAX 2500 X‐ray powder diffractometer equipped with a 18 kW Cu Kα radiation. The weight percent of elements was determined using an Oxford Instruments INCA EDS operating at an accelerating voltage of 15.0 kV. The optical image of the micropatterned piezoelectric layer was investigated by a Nikon ECLIPSE LV100ND optical microscope which was coupled with a charge‐coupled device camera. The ink rheology was measured using an Anton Paar Modular Compact Rheometer. A bending stage was utilized to apply a periodic deformation to the PNG at a desired displacement and speed. The electrical signal of the PNG device was obtained using a KEITHLEY Model 2450 Interactive SourceMeter SMU Instrument. The simulation investigation was conducted by using COMSOL multiphysics software.

## Supporting information

As a service to our authors and readers, this journal provides supporting information supplied by the authors. Such materials are peer reviewed and may be re‐organized for online delivery, but are not copy‐edited or typeset. Technical support issues arising from supporting information (other than missing files) should be addressed to the authors.

Supplementary
