# Supplementary material for: Direct Writing of Patterned, Lead‐Free Nanowire Aligned Flexible Piezoelectric Device
Source: Adv Sci (Weinh). 2016 May 30;3(8):1600120. doi: 10.1002/advs.201600120 (PMC5089621; doi:10.1002/advs.201600120)
Supplement: Supplementary file 1 — Supplementary [file ADVS-3-0q-s001.pdf]

Copyright WILEY-VCH Verlag GmbH & Co. KGaA, 69469 Weinheim, Germany, 2016.

ADVANCED SCIENCE
Open Access

Supporting Information

for *Adv. Sci.,* DOI: 10.1002/advs.201600120

Direct Writing of Patterned, Lead-Free Nanowire Aligned Flexible Piezoelectric Device

Meng Gao, Lihong Li, Wenbo Li, Haihua Zhou, and Yanlin Song**

Copyright WILEY-VCH Verlag GmbH & Co. KGaA, 69469 Weinheim, Germany, 2013.

Supporting Information

Direct Writing of Patterned, Lead-free Nanowire Aligned Flexible Piezoelectric Device

Meng Gao, Lihong Li, Wenbo Li, Haihua Zhou and Yanlin Song**

Synthesis of $(Na,K)NbO_3$ based material

Synthesis of $(Na,K)NbO_3$ nanowires

(1) Synthesis of KNb_3O_8 nanowires: Nb_2O_5 and KCl were mixed in ethanol in a mortar according to a molar ratio of 1:10. After dried at 120 °C, the mixture was heated in a furnace at 1000 °C for 3 h. The product was rinsed with deionized water to remove KCl salt afterwards.

(2) Synthesis of $H_3ONb_3O_8$ nanowires: 1.5 g of as-synthesized KNb_3O_8 was added to a 600 mL HNO_3 (2M) solution and mechanically stirred for 24 h at 150 °C. Then the product was washed with hot deionized water.

(3) Synthesis of Nb_2O_5 nanowires: the as-prepared $H_3ONb_3O_8$ was heated at 600 °C for 1 h.

(4) Synthesis of $(Na,K)NbO_3$ nanowires: the as-prepared Nb_2O_5 , K_2CO_3, Na_2CO_3 and KCl were mixed in the ratio of 1:0.45:0.55:10 in ethanol, and then heated at 850 °C for 10 min after dried at 120 °C.

Synthesis of $(Na,K)NbO_3$ nanoparticles

Nb_2O_5 , K_2CO_3, Na_2CO_3 and KCl were mixed in the ratio of 1:0.45:0.55:10 in ethanol, and then heated in a furnace at 850 °C for 10 min after dried at 120 °C.

Interrelation of the XRD Pattern and the Preferential Orientation

XRD pattern of the direct-written PNG device without top electrode shows a preferential orientation of KNN nanowires. According to the SAED pattern of the KNN nanowire in Figure 1d, the nanowire is single crystal with a growth direction of <010>, which is in agreement with previous report.[1] The diffraction peak (020) near 32° (2θ) which shows the plane perpendicular to the nanowire direction, demonstrates a decreased intensity compared with the XRD data of only KNN nanowires (Figure 1c). Thus, the XRD pattern verifies that nanowires in the direct-written device have lain down on the surface .

Relationship between strain variations and piezoelectric output

The piezoelectric constitutive equations which describe the mechanical and electrical conversion of piezoelectric materials can be explained as following: [2, 3]

$$S = s^E T + dE \quad (1)$$

$$D = dT + \varepsilon^T E \quad (2)$$

Where S is strain, T is the applied stress, D is the electric displacement, E is the electric field, sE is the elastic compliance at a constant electric field, εT is the permittivity at a constant stress, and d is the piezoelectric charge/strain coefficient. These equations explain the coupling between the mechanical and electric fields, which confirm that the strain variation produces different piezoelectric output.

Table S1. Electrical performance of previous reported lead-free PNGs

Piezoelectric materials	Innovative device feature	Output voltage [V]	References
ZnO	Hollow hemisphere structure	0.2	[4]
$BaTiO_3$	Nanoshell array	2.2	[5]
PVDF [a)]	Nanoporous array	2.6	[6]
P(VDF-TrFE) [b)]	Trigonal line-shaped and pyramid-shaped microstructures	4.4	[7]
$BaTiO_3$ nanoparticles and P(VDF-HFP) [c)]	Hemispherically aggregated microstructures	75	[8]
P(VDF-TrFE)	Aligned arrays of nanofibers	1.5	[9]

ZnO nanowires	Aligned arrays of nanowires	2.03	[10]
$BaTiO_3$ film	Highly oriented nanoplatelets	6.5	[11]
P(VDF-TrFE)	Highly sensitive stretchable device	8	[12]
PVDF–$NaNbO_3$ nanofiber	All-fiber device	3.4	[13]
$BaTiO_3$ nanoparticles	Graphitic carbons serve as filler	3.2	[14]
$0.942(K_{0.480}Na_{0.535})NbO_3$-$0.058LiNbO_3$ nanoparticles	Copper nanorods serve as filler	12	[15]
$BaTiO_3$ nanoparticles	Bacterial cellulose serves as matrix	14	[16]
$ZnSnO_3$ nanocubes	High piezoelectric coefficient of the piezoelectric material	20	[17]
Li-doped ZnO nanowires	High piezoelectric coefficient of the piezoelectric material	30	[18]

[a)] PVDF is the abbreviation of polyvinylidene fluoride; [b)] P(VDF-TrFE) is the abbreviation of poly(vinylidene fluoride-co-trifluoroethylene); [c)] P(VDF-HFP) is the abbreviation of poly-(vinylidene fluoride-co-hexafluoropropene)

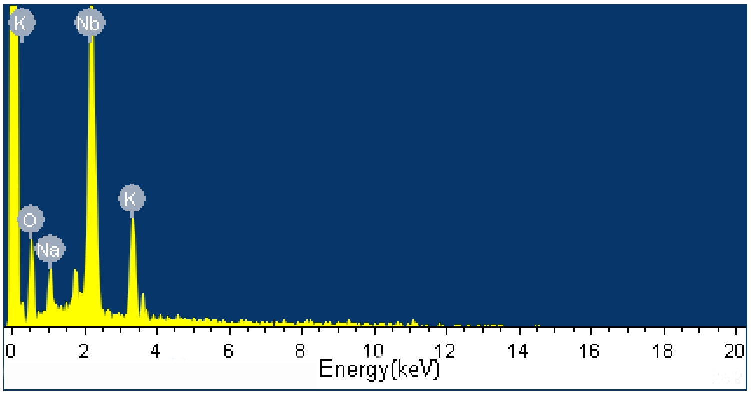

Figure S1 EDS result of the KNN NWs.

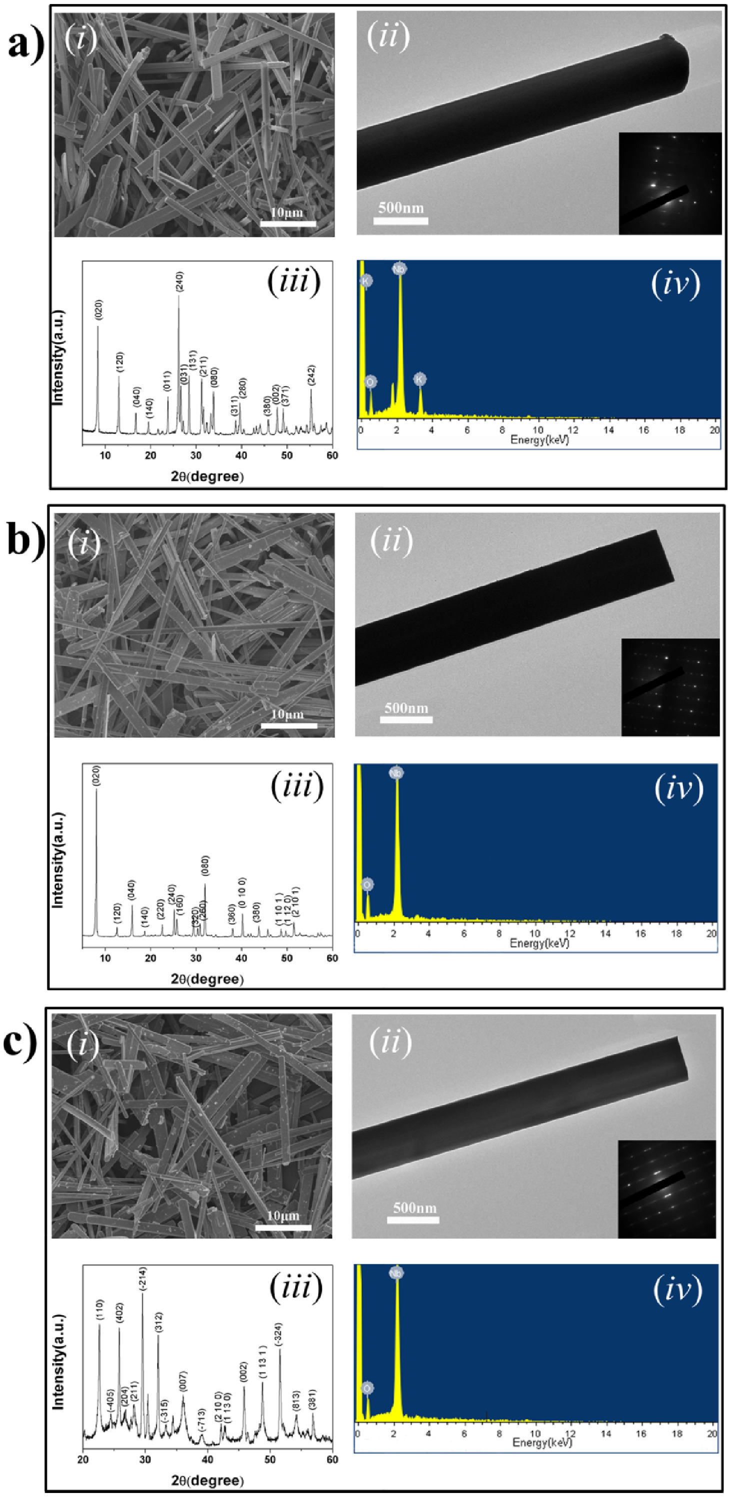

Figure S2 The morphology, crystalline property, element distribution characterization of (a) KNb_3O_8, (b) $H_3ONb_3O_8$ and (c) Nb_2O_5, the intermediate products of the KNN NWs. (*i*), (*ii*), (*iii*), (*iv*) are SEM photograph, TEM image, XRD pattern and EDS result, respectively. The inset shows the corresponding SAED pattern.

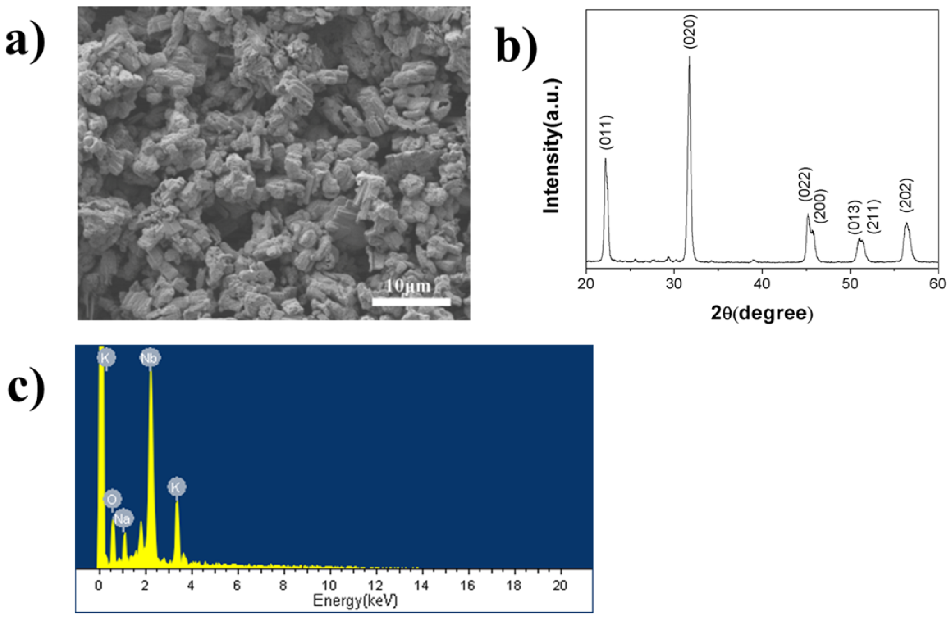

Figure S3 Characterization of the KNN NPs, (a) SEM image, (b) XRD pattern, (c) EDS result.

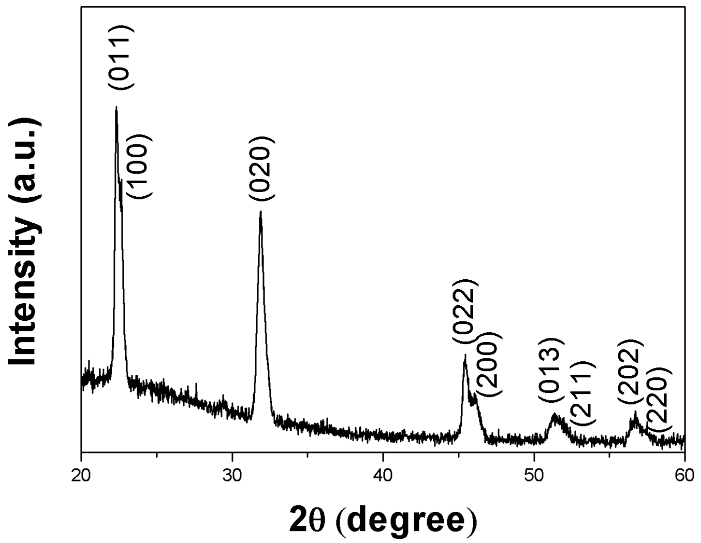

Figure S4 XRD pattern of the NWs-DW PNG without top electrode.

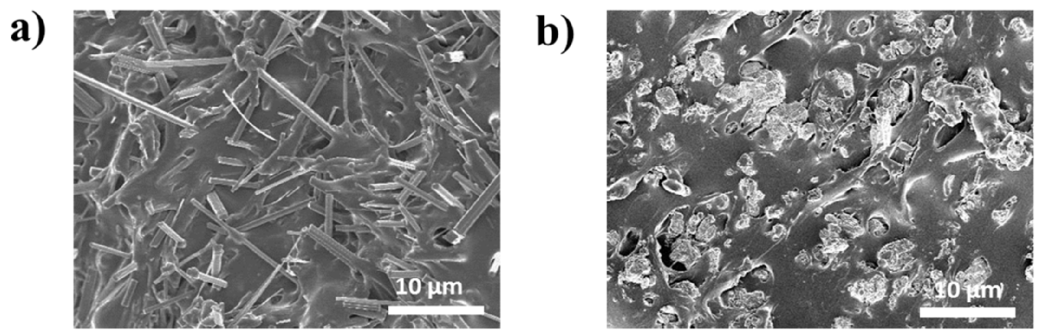

Figure S5 (a) Cross-sectional SEM photographs of the piezoelectric layer of (a) the NWs-SC PNG and (b) the NPs-SC PNG.

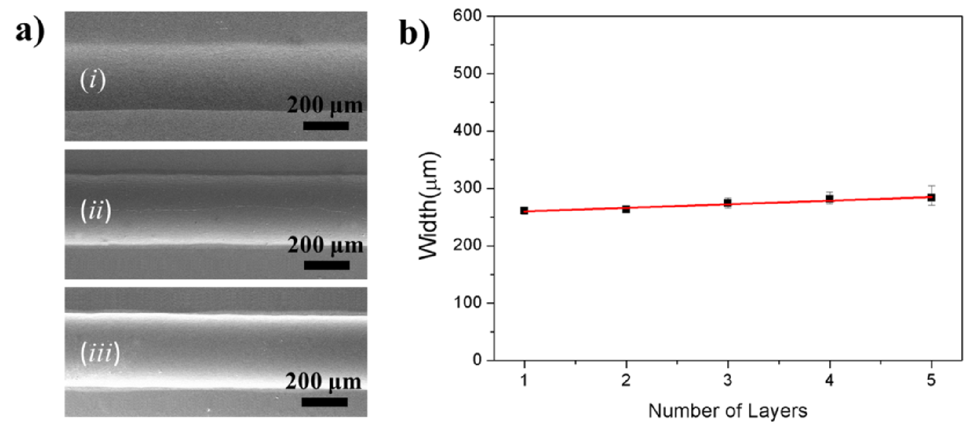

Figure S6 (a) Top SEM images of the printed (*i*) 1 layer, (*ii*) 3 layers, (*iii*) 5 layers piezoelectric structure, (b) Printed width features as a function of the printed layer number (200 μm nozzle diameter).

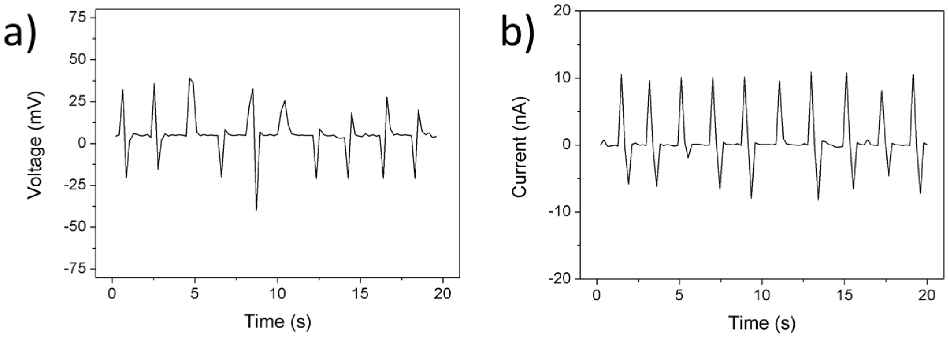

Figure S7 (a) Voltage output and (b) current output of the control device fabricated by direct writing the PDMS matrix without the KNN NWs.

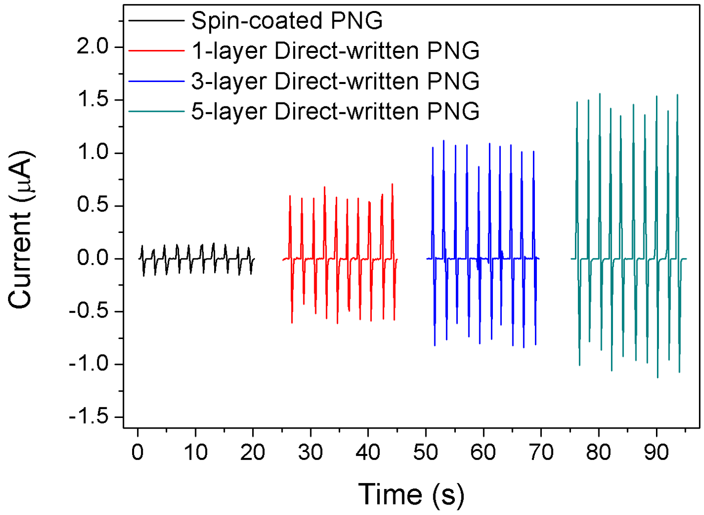

Figure S8 Current output of a NWs-SC PNG and NWs-DW PNGs with different layers.

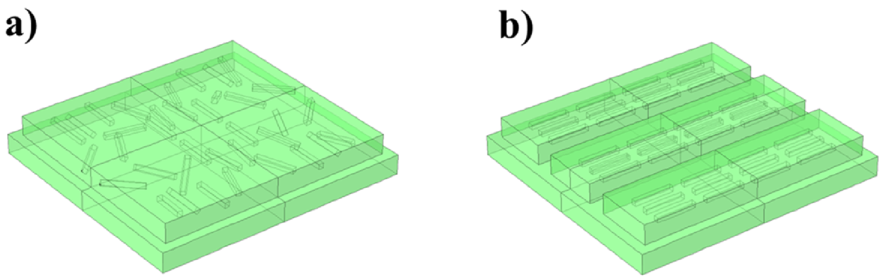

Figure S9 Half transparent morphology images corresponded to the voltage distribution simulation of (a) the NWs-SC PNG and (b) the NWs-DW PNG, respectively.

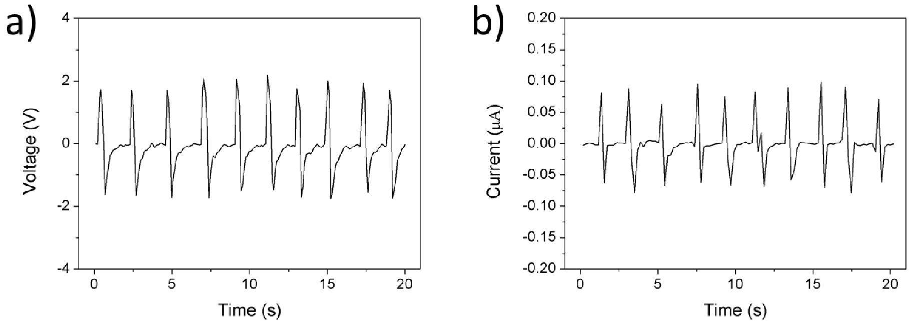

Figure S10 (a) Voltage output and (b) current output of the NPs-SC PNG.

References

[1] L.-Q. Cheng, K. Wang, J.-F. Li, *Chem. Commun.* **2013**, *49*, 4003.
[2] Z. Wang, X. Pan, Y. He, Y. Hu, H. Gu, Y. Wang, *Adv. Mater. Sci. Eng.* **2015**, *10*, 1155.
[3] Y. Qi, M. C. McAlpine, *Energy Environ. Sci.* **2010**, *3*, 1275.
[4] J. Chun, K. Y. Lee, C.-Y. Kang, M. W. Kim, S.-W. Kim, J. M. Baik, *Adv. Funct. Mater.* **2014**, *24*, 2038.
[5] M.-L. Seol, H. Im, D.-I. Moon, J.-H. Woo, D. Kim, S.-J. Choi, Y.-K. Choi, *ACS Nano* **2013**, *7*, 10773.
[6] S. Cha, S. M. Kim, H. Kim, J. Ku, J. I. Sohn, Y. J. Park, B. G. Song, M. H. Jung, E. K. Lee, B. L. Choi, J. J. Park, Z. L. Wang, J. M. Kim, K. Kim, *Nano Lett.* **2011**, *11*, 5142.
[7] J.-H. Lee, H.-J. Yoon, T. Y. Kim, M. K. Gupta, J. H. Lee, W. Seung, H. Ryu, S.-W. Kim, *Adv. Funct. Mater.* **2015**, *25*, 3203.
[8] S.-H. Shin, Y.-H. Kim, M. H. Lee, J.-Y. Jung, J. Nah, *ACS Nano* **2014**, *8*, 2766.
[9] L. Persano, C. Dagdeviren, Y. Su, Y. Zhang, S. Girardo, D. Pisignano, Y. Huang, J. A. Rogers, *Nat. Commun.* **2013**, *4*, 1633.
[10] G. Zhu, R. Yang, S. Wang, Z. L. Wang, *Nano Lett.* **2010**, *10*, 3151.
[11] T. Gao, J. Liao, J. Wang, Y. Qiu, Q. Yang, M. Zhang, Y. Zhao, L. Qin, H. Xue, Z. Xiong, L. Chen, Q.-m. Wang, *J. Mater. Chem. A* **2015**, *3*, 9965.
[12] J.-H. Lee, K. Y. Lee, B. Kumar, T. Nguyen Thanh, N.-E. Lee, S.-W. Kim, *Energy Environ. Sci.* **2013**, *6*, 169.
[13] W. Zeng, X.-M. Tao, S. Chen, S. Shang, H. L. W. Chan, S. H. Choy, *Energy Environ. Sci.* **2013**, *6*, 2631.
[14] K.-I. Park, M. Lee, Y. Liu, S. Moon, G.-T. Hwang, G. Zhu, J. E. Kim, S. O. Kim, D. K. Kim, Z. L. Wang, K. J. Lee, *Adv. Mater.* **2012**, *24*, 2999.
[15] C. K. Jeong, K.-I. Park, J. Ryu, G.-T. Hwang, K. J. Lee, *Adv. Funct. Mater.* **2014**, *24*, 2620.
[16] G. Zhang, Q. Liao, Z. Zhang, Q. Liang, Y. Zhao, X. Zheng, Y. Zhang, *Adv. Sci.* **2016**, *3*, 1500257.
[17] K. Y. Lee, D. Kim, J.-H. Lee, T. Y. Kim, M. K. Gupta, S.-W. Kim, *Adv. Funct. Mater.* **2014**, *24*, 37.
[18] S.-H. Shin, Y.-H. Kim, M. H. Lee, J.-Y. Jung, J. H. Seol, J. Nah, *ACS Nano* **2014**, *8*, 10844.
